# Encoding the pitch of sounds using synchrony receptive fields

**DOI:** 10.1186/1471-2202-12-S1-P21

**Published:** 2011-07-18

**Authors:** Jonathan Laudanski, Romain Brette

**Affiliations:** 1 Laboratoire Psychologie de la Perception, CNRS and Université Paris Descartes, 45, rue des Saints Pères, 75006 Paris, France; 2 Equipe Audition, Département d'Etudes Cognitives, Ecole Normale Supérieure, 29, rue d'Ulm, 75005 Paris, France

## 

Pitch constitutes a major dimension of auditory perception along which periodic (or near-periodic) sounds can be organized. Although the role of pitch is essential in the perception of music and important when dealing with source segregation in complex auditory scene, the neural mechanisms underlying pitch perception remain unclear [1]. Many models have been proposed including models relying purely on rate-based strategy and filters selectivity along the tonotopic axis, or models relying on the accurate timing of spikes [2] along delay lines, as well as intermediate models [3] using both the tonotopic and temporal organization of auditory nerve activity (for a review see [4]).

We propose here a model of pitch perception based on the synchrony produced within groups of peripheral neurons. We first describe how auditory stimuli can induce for peripheral neurons synchronous outputs. This stimulus-based synchrony is independent of the spiking neuron model and can thus be used to define two dual concepts [5]: 1) the synchrony receptive field (SRF) of a group of neurons and 2) the synchrony partition of a peripheral neural population. We describe how periodic sounds induce specific partitions of the peripheral neural population and how these partitions can be used to encode sound periodicity. Hence, we show how to use neural assemblies to encode a sound’s pitch. We demonstrate our theory using hard-wired networks of integrate-and-fire neurons making synapses onto a population of coincidence detectors. Using the RWC music database, we recover the pitch of a large variety of sounds, including different voices and musical instruments. We illustrate how our synchrony-based scheme encodes pitch independently of sound intensity, or type of source. We detail how mode-locking can pose issues when periodically forcing the spiking peripheral neurons and show how these issues can be handled. Finally, we discuss the extent of overlap between previous models of pitch and our scheme, highlighting the interest of SRFs with respect to the biological plausibility of the emergence of such synchronous neural assemblies.
